# Non-Contact Monitoring of Fetal Movement Using Abdominal Video Recording

**DOI:** 10.3390/s23104753

**Published:** 2023-05-15

**Authors:** Qiao Han, Dongmei Hao, Lin Yang, Yimin Yang, Guangfei Li

**Affiliations:** 1 Faculty of Environment and Life, Beijing University of Technology, Beijing 100124, China; 2 Beijing International Science and Technology Cooperation Base for Intelligent Physiological Measurement and Clinical Transformation, Beijing 100124, China

**Keywords:** fetal movement, non-contact, optical flow, video

## Abstract

Fetal movement (FM) is an important indicator of fetal health. However, the current methods of FM detection are unsuitable for ambulatory or long-term observation. This paper proposes a non-contact method for monitoring FM. We recorded abdominal videos from pregnant women and then detected the maternal abdominal region within each frame. FM signals were acquired by optical flow color-coding, ensemble empirical mode decomposition, energy ratio, and correlation analysis. FM spikes, indicating the occurrence of FMs, were recognized using the differential threshold method. FM parameters including number, interval, duration, and percentage were calculated, and good agreement was found with the manual labeling performed by the professionals, achieving true detection rate, positive predictive value, sensitivity, accuracy, and F1_score of 95.75%, 95.26%, 95.75%, 91.40%, and 95.50%, respectively. The changes in FM parameters with gestational week were consistent with pregnancy progress. In general, this study provides a novel contactless FM monitoring technology for use at home.

## 1. Introduction

Fetal movement (FM) has long been used as an important indicator of fetal health and neurobehavioral development [1]. The assessment of FM is an accepted method of identifying adverse pregnancy outcomes, including intrauterine growth restriction [2], oligohydramnios [3], and stillbirth. Some examples of FM include general body movements, kicks, stretches, rotation, twitch movements, limb movements, etc. [4]. There is normally a variation in FM, with a wide range in the number of movements per hour [5]. Studies have shown that medical interventions during the period of FM decrease may result in the delivery of a healthy, living baby [6,7]. In addition to reduced FM, excessive FM can be a risk factor contributing to stillbirth [8]. Therefore, early detection of risk factors and timely intervention can reduce the incidence of stillbirth by establishing prenatal FM detection.

Self-counting FM at home in a state which is calm and stable for pregnant women is economical and convenient for monitoring FM during pregnancy. FM is normally first perceived by the mother between 18 and 20 weeks of gestation. However, the sensitivity of pregnant women to FM varies widely [9], and the long-term monitoring of FM through subjective judgments is challenging. Only 37–88% of FMs are reported to be felt with the mother lying still and actively paying attention. In other cases, the actual frequency of FM and the mother’s ability to perceive these movements are influenced by many factors, such as the mother’s activity, stress, position, and attention level [10].

Ultrasound technology can visually assess the health of a fetus and is the most widely clinical method of identifying FM [11]. However, this technique requires an experienced clinician to operate, and the prolonged use of ultrasound may cause potential harm to the fetus [12,13,14]. Advances in fetal magnetic resonance imaging (MRI) through cine-MRI scans allow for the direct monitoring of the movements of the entire fetus [15]. However, this technique is expensive and has limited accessibility, and is mostly used in clinical settings. Moreover, this technique is not suitable for use in the continuous and prolonged monitoring of FM due to its large scale and operational complexity and due to the health risks of accumulative exposure [14].

In the current field of FM signal measurement based on wearable sensors, various transducers are placed on the abdominal wall to detect FM, including piezoelectric films for pressure [16], strain gauges for force [17], capacitive [18] or inductive [19] moving elements for deflection, and optical fibre for strain [4], etc. These measurements have the advantage of capturing automated and longitudinal data in the out-of-hospital setting. However, multiple transducers and multichannel signal processing must be used to eliminate signal noise from non-FM sources. The compliance matching between the transducer and the abdominal wall is vital to obtain reliable signals. The tightness of the strap holding these transducers in place possibly impairs the FM measurement. Additionally, electrocardiographic (ECG)-based FM tracking has also been proposed, including temporal and spatial ECG shape identification and fetal vectorcardiogram (VCG) loop alignment [20]. However, the multi-channel measurements and complex signal processing techniques required for maternal ECG removal and fetal VCG loop calibration make FM detection challenging.

Currently, many methods have been proposed for non-contact measurement. The camera system can monitor vital signs through the use of RGB cameras, IR cameras, and depth cameras. With algorithms for the post-processing of acquired video data, the heart and breathing rates are obtained unobtrusively and comfortably in both adults and neonates [21,22,23]. Motion information from optical flow has been applied to the diagnosis of neonatal seizures [24]. The method considers the pixel areas with velocities over a predetermined threshold to determine whether or not the detected movement matches the profile of a neonatal seizure. Koolen et al. [25] detected the respiration rate from the neonatal video included in polysomnography. Eulerian video amplification was used to amplify respiration motion, and optical flow algorithms were used to estimate respiration motion and obtain respiration signals. The respiration rate was successfully determined for sleeping-stage patients. Yue Sun et al. investigated an automated pipeline to estimate respiration signals of preterm infants in the neonatal intensive care units (NICUs) from video using the optical flow methods [26]. The conventional optical flow estimation method was compared with the deep learning-based flow estimation method to estimate the pixel motion vectors between adjacent frames. The experimental results contributed to furthering research into and the clinical applications of respiration monitoring methods via video.

This paper aims to propose an unobtrusive and non-contact method for the detection of FM using abdominal videos recorded by a camera. In our method, we used the optical flow algorithm, as well as image and signal processing techniques, for FM recognition and FM parameter calculation. Then, a few videos from pregnant women were collected to verify the feasibility of our study.

## 2. Materials and Methods

**Figure 1:**
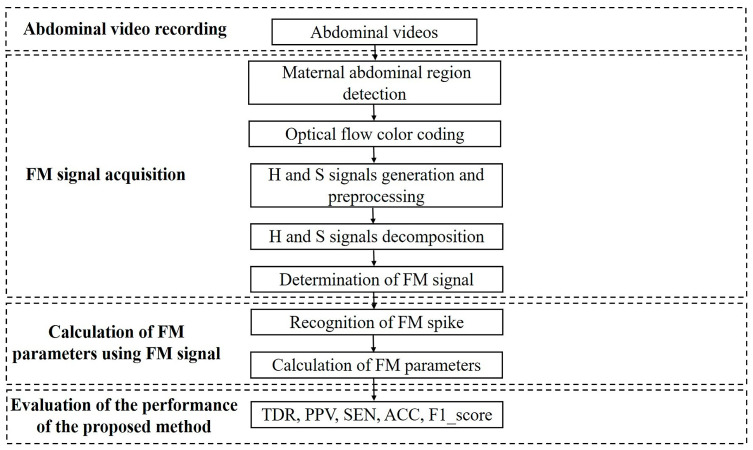
Flow chart of the proposed method.

The overall flow chart of the proposed method is shown in Figure 1, including stages of abdominal video recording, FM signal acquisition, FM parameter calculation, and the evaluation of the performance of the proposed method. In brief, the abdominal videos were recorded, and maternal abdominal regions were detected in each frame. Then, the optical flow vector of the abdomen was obtained and color-coded with hue (H) and saturation (S). Ensemble empirical mode decomposition (EEMD), energy ratio, and correlation analysis were applied to the H and S signals to determine the FM signal. Next, FM spikes were recognized using the differential threshold method, and FM parameters were calculated. Finally, the performance of the proposed method was evaluated regarding the manually labeled results.

### 2.1. Abdominal Video Recording

A total of 5 pregnant women participated in this study during 28 to 36 gestational weeks (GWs). When they felt distinct FM, they stayed in bed, quietly exposing their abdomens to the camera, and recorded abdominal videos for approximately 40 min. To achieve high-quality images and a complete FM capture, we used the digital image acquisition system (Spedal MF934H, Shenzhen New color Creative Electronics Ltd., Shenzhen, China) with a resolution of 1280 × 720 and a frame rate of 25 frames/s. A total of 18 abdominal videos were obtained. Of the videos recorded, two poor-quality abdominal videos were excluded, and the other 16 abdominal videos were analyzed to validate our proposed method. The subjects were asked to sign a consent agreement after being informed of the study’s aim, potential benefits, and risks. The study was approved by the Ethics Committee of Science and Technology of Beijing University of Technology and was conducted according to the specifications of the Declaration of Helsinki of the World Medical Association.

### 2.2. Fetal Movement Signal Acquisition

To automatically measure FM from the recording abdominal videos, we first acquired the FM signal using the following five steps.

#### 2.2.1. Maternal Abdominal Region Detection

**Figure 2:**
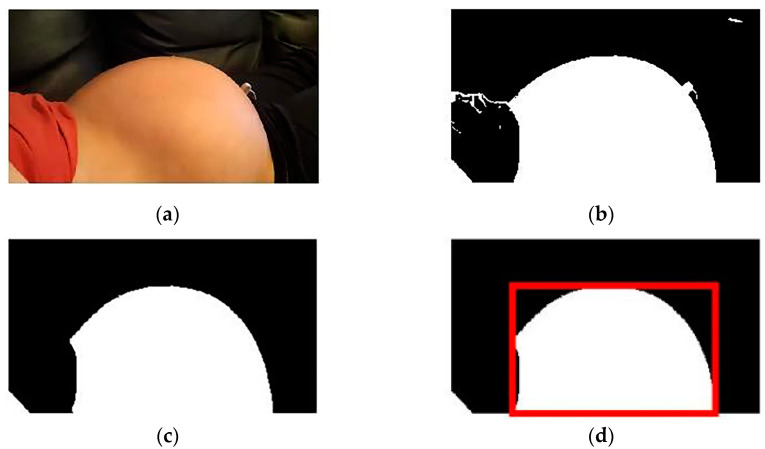
The process of abdominal region detection on one frame. (**a**) The original image, (**b**) the detected skin region, (**c**) the image after the open operation, and (**d**) the positioning of the abdominal region.

The maternal abdomen had to be positioned in each frame for the detection of FM. Firstly, the frames with maternal movements were deleted manually. Then, skin regions were extracted using the ellipse skin model [27] to exclude non-skin regions as far as possible, and the disconnected skin regions were eliminated by the open operation. Subsequently, the eight connected domains were calculated, and the abdominal candidate regions were selected. If the aspect ratio of the candidate region was larger than 1, the region was regarded as the abdominal region. Figure 2 shows the process of abdominal region detection on one frame of an image.

#### 2.2.2. Optical Flow Color-Coding

We used the change in optical flow to represent the rise and fall in the maternal abdomen caused by FM. The optical flow field is a vector field that expresses the kinematic relationship between local 2D or 3D images [28]. Optical flow algorithms use the spatiotemporal patterns of the images or signals to estimate the motion field.

**Figure 3:**
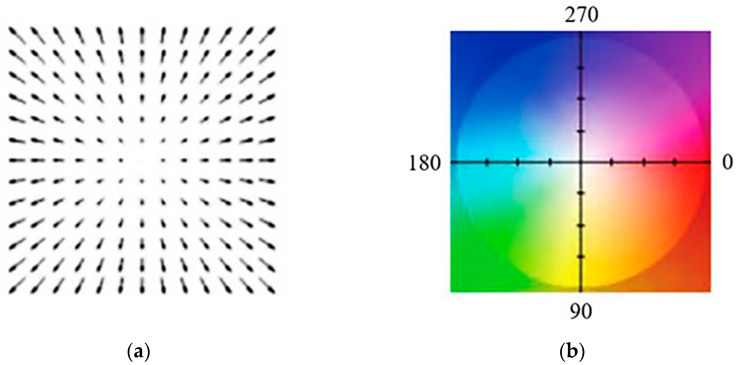
The optical flow visualization, (**a**) the optical flow vector; (**b**) the color-coding of the optical flow.

**Figure 4:**
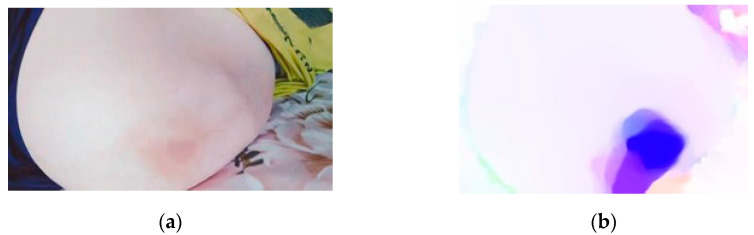
The optical flow color-coding of FM. (**a**) The *k*^th^ frame, and (**b**) the *k*^th^ frame optical flow color-coded image.

The velocity vector of each pixel in the image was obtained using the optical flow method [29]. The direction and magnitude of the velocity vector of each pixel were represented by hue (H) and saturation (S) in an HSL color space, respectively. The visualized optical flow is shown in Figure 3. Figure 3a represents the optical flow vector, the optical flow vector for each pixel being a vector from the center of the square to that pixel; Figure 3b represents the color-coding of the optical flow. The stationary points in the image are white: the darker the color, the greater the magnitude of the optical flow. Figure 4 shows the *k*^th^ frame of the abdominal video after optical flow color-coding. The most obvious dark area in Figure 4b indicates the FM to the upper left. In this instance, the darker the color, the greater the amplitude of the FM. Conversely, the other areas are close to white, indicating these areas are stationary.

#### 2.2.3. H and S Signals Generation and Preprocessing

H and S channels in the *k*^th^ frame image were obtained from the abdominal video images using the following formula.

(1) $$R(k)=\frac{1}{N}\sum_{i=1}^{N}\theta_{i}$$

where R(k) is the H or S channel of the k^th^ frame image, *N* is the number of pixels, and $\theta_{i}$ is the i^th^ pixel value of the H or S channel within the abdominal region.

FM frequency was within the range of 3 to 20 Hz [30] and the highest frequency of the H and S signal time series did not exceed 20 Hz. Therefore, a 7th-order Butterworth high-pass filter with a cutoff frequency of 3 Hz was designed to remove the low-frequency noise of the H and S signal time series.

#### 2.2.4. H and S Signals Decomposition

We decomposed the H and S signals into a finite number of intrinsic mode functions (IMFs) to further remove the high-frequency interference after preprocessing. Being non-linear and non-stationary, H and S signals can be decomposed according to their timescale characteristics using the conventional empirical mode decomposition (EMD) algorithm. However, a major drawback of the EMD algorithm is mode mixing, i.e., a single IMF either consisting of signals of widely disparate sizes or a similarly sized signal residing in a different IMF component. To overcome the scale separation problem, the present study employed the EEMD algorithm [31], which defined the true IMF components as the average of an ensemble of trials, each being composed of a signal and a white noise. The white noise was added to the H and S signals, and the different timescale components were mapped to the reference timescale associated with the white noise. Meanwhile, the white noise would be eliminated by using multiple averaging and using the ensemble average as the component of the signal.

In this study, the ratio of the standard deviation between the white noise and the S signal (or H signal) was set to 0.1 [32], and the ensemble number of the EEMD algorithm was set to 50.

**Figure 5:**
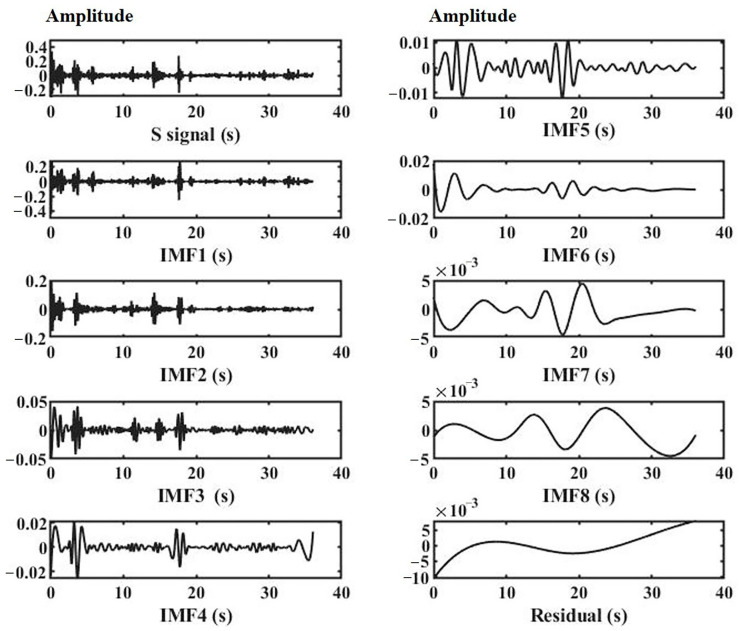
The IMFs (IMF1 to IMF8) and the residual of the S signal.

Figure 5 shows an example where the S signal was decomposed into eight IMFs and one residual. Here, IMF1 represents the highest frequency and IMF8 represents the lowest frequency among them.

#### 2.2.5. Determination of Fetal Movement Signal

FM signal was determined by combining the energy ratio and Spearman correlation coefficient. The energy ratio was calculated as follows: for the IMF*_j_* (*j* = 1, 2, …, m), the energy ratio of FM component *E_f_*(*j*) (3 to 20 Hz) to the total energy *E*(*j*) of IMF*_j_* (*j* = 1, 2, …, m) was calculated. *E_f_*(*j*) and *E*(*j*) were obtained using the fast Fourier transform algorithm. For the IMF*_j_*, if *E_f_*(*j*)/*E*(*j*) > δ, the IMF*_j_* was supposed to be associated with FM. The *δ* of 0.6 was set, which was a trade-off between information loss and interference introduction.

The Spearman correlation coefficient η was calculated to describe both linear correlation and non-linear correlation between these two time series. The calculation formula used is as follows:

(2) $$\eta =\frac{\sum_{i=1}^{n}\left(x_{i}-\overset{-}{x})(y_{i}-\overset{-}{y}\right)}{\sqrt{\sum_{i=1}^{n}{\left(x_{i}-\overset{-}{x}\right)}^{2}}\sqrt{\sum_{i=1}^{n}{\left(y_{i}-\overset{-}{y}\right)}^{2}}}$$

where *n* is the sample size of the data, $x_{i}$ is the rank of i^th^ point of IMF*_j_* (*j* = 1, 2, …, m)$,y_{i}$ is the i^th^ point of H signal (or S signal). $\overset{-}{x}$ and $\overset{-}{y}$ are the mean of IMF*_j_* (*j* = 1, 2, …, m) and the H signal (or S signal), respectively.

The Spearman correlation coefficient between IMF*_j_* (*j* = 1, …, m) and the H signal (or S signal) was calculated separately. Finally, the IMF*_j_* (*j* = 1, 2, …, m) with the maximum of (*E_f_*(*j*)/*E*(*j*) + η) was selected as the FM signal.

### 2.3. Calculation of Fetal Movement Parameters Using Fetal Movement Signal

#### 2.3.1. Recognition of Fetal Movement Spike

**Figure 6:**
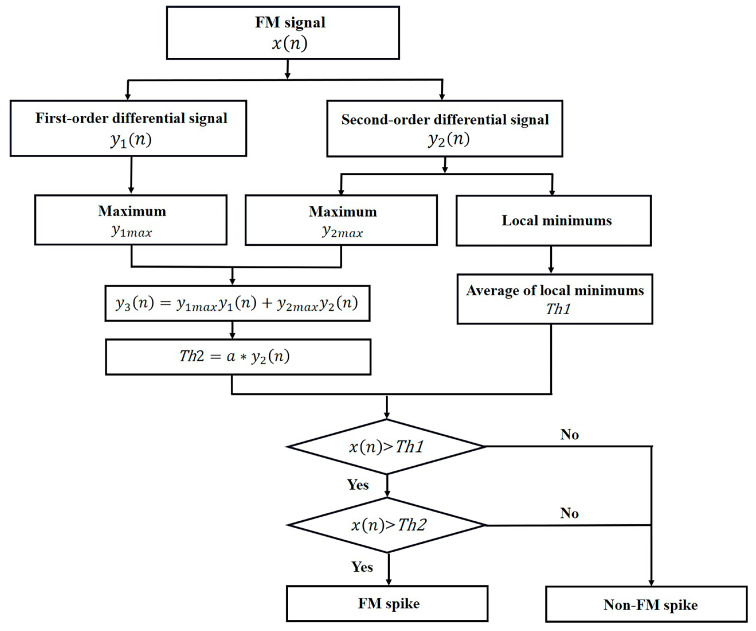
Flow chart of FM spike recognition.

FM usually causes the FM signal to change dramatically. Therefore, this study combined the first-order and second-order difference in FM signal to determine FM spike, representing the occurrence of FM. The flow chart for FM spike recognition is shown in Figure 6.

For the FM signal x(n), its first-order difference $y_{1}\left(n\right)$ and second-order difference $y_{2}\left(n\right)$ were calculated as (3) and (4), respectively.

(3) $$y_{1}\left(n\right)=x\left(n+1\right)-x(n)$$

(4) $$y_{2}\left(n\right)=x\left(n+2\right)-2x(n+1)+x(n)$$

The local minima of the second-order difference were averaged as the first threshold Th1. If x(n) ≤ Th1, then it was a non-FM spike; otherwise, it would be further recognized by the second threshold Th2.

The second threshold Th2 was determined with (5) and (6).

(5) $$y_{3}\left(n\right)=y_{1max}y_{1}\left(n\right)+y_{2max}y_{2}\left(n\right)$$

(6) $$Th2=a*y_{3max}$$

where $y_{1max}$, $y_{2max}$, and $y_{3max}$ are the maxima of $y_{1}\left(n\right)$, $y_{2}\left(n\right)$, and $y_{3}\left(n\right)$, respectively. a is a coefficient between 0.01 and 0.05.

If $x\left(n\right)>Th1$ and $x\left(n\right)>Th2$, it was determined as an FM spike.

#### 2.3.2. Calculation of Fetal Movement Parameters

FM parameters including number, interval, duration, and percentage were calculated to describe the FM characteristics. For the FM number, we highlighted that if the interval between two adjacent FM spikes was less than 6 s [33], they were regarded as one FM. The FM number per hour was deduced in this way. The FM interval was defined as the elapsed time between two adjacent FMs. The FM duration referred to the time interval between two adjacent spikes less than 6 s [34,35,36]. The FM percentage was the total FM duration as a percentage of the recording time.

**Figure 7:**
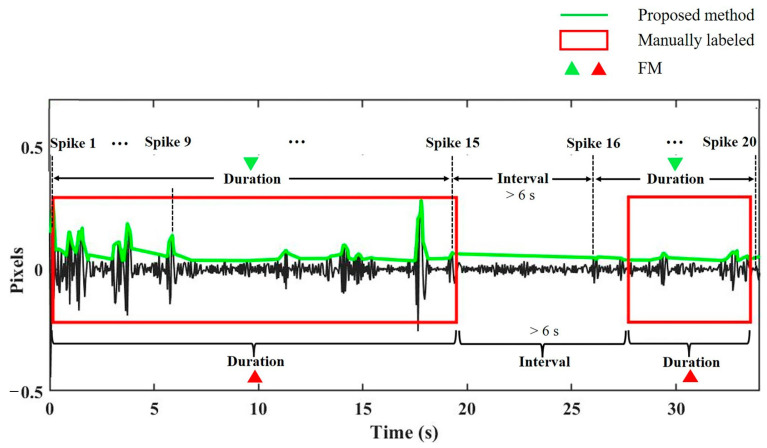
The FMs recognized by the proposed method and manually labeled. The red rectangle indicates a manually labeled FM, with its left and right edges corresponding to the start and end frames of the FM, respectively. The black indicates the FM signal, and the green indicates the FM spikes recognized by the proposed method.

As shown in Figure 7, the red rectangle represents an FM manually labeled by two professionals on the abdominal video as a gold standard, with its length indicating the FM duration. All the green spikes within the red rectangle were regarded as one FM because the interval between any two adjacent spikes was less than 6 s.

### 2.4. Evaluation of the Performance of the Proposed Method

FM that had been manually labeled by the professionals on the abdominal video was used as the gold standard. The performance of the proposed method is expressed in terms of true detection rate (TDR), positive predictive value (PPV), sensitivity (SEN), accuracy (ACC), and F1_score as follows:

(7) $$TDR=\frac{TP}{TME}\times 100$$

(8) $$PPV=\frac{TP}{\left(TP+FP\right)}\times 100$$

(9) $$SEN=\frac{TP}{\left(TP+FN\right)}\times 100$$

(10) $$ACC=\frac{TP}{\left(TP+FP+TN\right)}\times 100$$

(11) $$F1\_score=\frac{2\times PPV\times SEN}{\left(PPV+SEN\right)}\times 100$$

where TME is the number of FM manually labeled by the professionals, TP (true positive) is the number of FM that was correctly recognized, FP (false positive) and FN (false negative) are the number of FM that were falsely recognized with the proposed method separately.

In addition, Bland–Altman analysis was utilized to assess the agreement of FM parameters between the proposed method and the FMs manually labeled by the professionals.

## 3. Results

### 3.1. Comparison of the Detection Result

Table 1 shows the FM parameters, including FM number, interval, duration, and percentage detected by the proposed method with the H signal and S signal in comparison to the results obtained via manually labeling by professionals. As can be seen from Table 1, the FM parameters measured with the S signal are closer to the manually labeled results.

We evaluated the detection results with the H signal and S signal, respectively. As shown in Table 2, the S signal has a better performance than the H signal.

### 3.2. Bland–Altman Analysis of Fetal Movement Parameters

**Figure 8:**
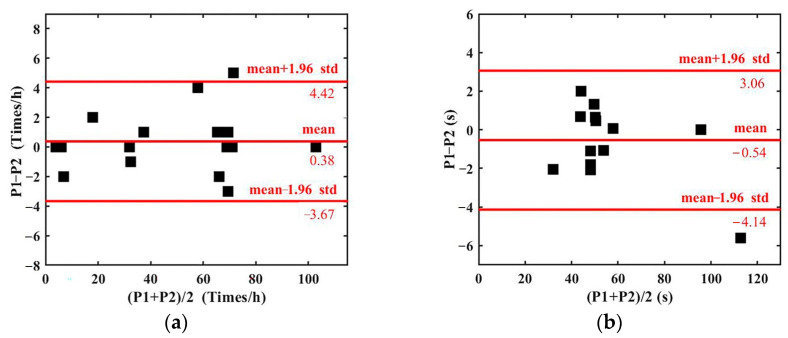

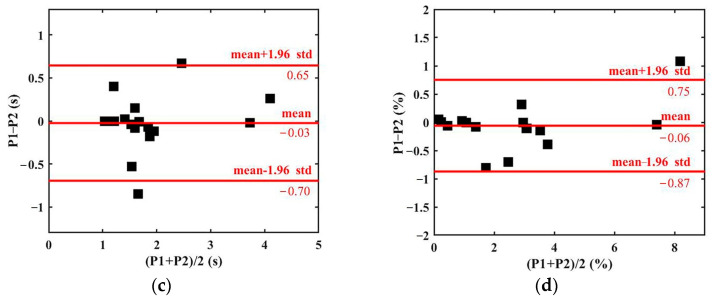
Bland–Altman analysis of FM parameters detected by the proposed method (P1) and the manually labeled (P2). (**a**) FM number; (**b**) FM interval; (**c**) FM duration; (**d**) FM percentage.

Bland–Altman analysis was utilized to assess the agreement of FM parameters between the proposed method and the manually labeled by the professionals. Figure 8 shows the mean, difference, and 95% limits of FM parameters.

Figure 8 indicates that the mean of the difference is small, that the differences are mostly within a 95% confidence interval and that therefore there is good agreement with the manually labeled FM parameters.

### 3.3. Comparison of FM Parameters between Gestational Weeks

**Figure 9:**
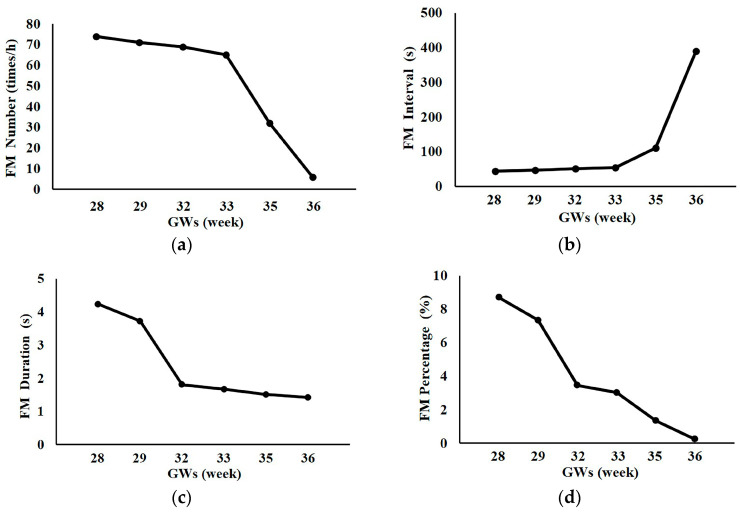
Comparison of FM parameters between different GWs. (**a**) FM number; (**b**) FM interval; (**c**) FM duration; (**d**) FM percentage.

Figure 9 shows the FM parameters obtained from different GWs in one pregnant woman. It was noticed that FM number, duration, and percentage decreased, while FM interval increased, with gestational week.

## 4. Discussion

FM is considered to be one of the fundamental manifestations of early neural activity because it is spontaneously generated by the central nervous system. FM helps the clinician understand the functional development of the fetus. Active fetal monitoring methods, such as ultrasound techniques, are expensive and there are objections to their long-term use. Maternal perception is unreliable. Passive fetal monitoring methods, such as accelerometry and electrodes placed on pregnant women, are still not accurate. This study presented a novel method to measure FM continuously using a camera to record abdominal video without touching the pregnant women and thus without inducing any inconvenience to them.

Table 3 summarizes the performance of the abdominal video-based FM detection in this study in terms of TDR, PPV, SEN, ACC, and F1_score in comparison with the previously published papers. Most of the related studies evaluated their results with ultrasound or maternal perception. However, abdominal video recording and the operation of ultrasound equipment could not be performed simultaneously in our study. Maternal perception is known to be inaccurate and unable to provide FM parameters. Therefore, two professionals carefully watched the abdominal videos and labeled the FM’s start and end frames, which were then used as the gold standard in our evaluation. Although a direct comparison was not feasible due to the difference between the database and clinical scenario, our proposed method generated promising results.

Besides FM number, our proposed method also provided FM interval, duration, and percentage. These parameters provide obstetricians with more information about FM and help them to identify risks during pregnancy. To the best of our knowledge, no other study has present these FM parameters.

In particular, we followed one pregnant woman from 28 to 36 GWs and found that her FM number, duration, and percentage decreased, while her FM interval increased with gestational week. These results in line with the related studies [40,41]. These changes might be due to the increasing conservation of energy in preparation for childbirth, as well as the reduction in uterine space [42]. Ten suggested that the overall decline in the incidence of FM during pregnancy appeared to be a fetal developmental phenomenon [43]. Ryo proposed that the period of no FM means fetal stillness, and that the increase in the period without FM might be a sign that the development of the central nervous system was gaining control over the peripheral nervous system and reducing body movements [44]. Nijhuis et al. [45] reported that the fetal behavioral state was not fully established until 36 weeks. Before 36 weeks, rapid changes in FM may be associated with the development of the fetal behavioral state.

This study proposed a non-contact FM measurement based on abdominal video, which is simple and suitable for home health care, and thus the FM parameters acquired could be transferred to the clinicians over the Internet. Regarding the privacy of the subjects, the proposed method did not pose any privacy issues compared to other video-based methods that used frontal images (including the face of the subject). This was because the camera only captured the subject’s abdomen and did not show the identifiable parts of the subject. As far as we know, no such method has been introduced for FM detection in other studies. Previously, various sensors required contact with the skin of pregnant women, which increased the risk of skin irritation. Sensor fixing straps influenced the subjects’ normal breath and even normal FM since the bands were tightly tied to the abdomen, resulting in the unnatural activity of the abdomen [46]. While one or more sensors were attached to the skin of pregnant women’s abdomen using medical-grade adhesive patches [47,48], prolonged contact also had the ability to cause discomfort. However, the proposed method has the potential to have a better performance in practical applications and be more comfortable. Therefore, this is a viable option for the long-term monitoring of FM, designed to detect early reductions in FM while reducing the efforts of expectant mothers who would otherwise have to actively count the number of FM daily. The proposed monitoring method was convenient and had good adaptability.

Furthermore, the ordinary camera could be used to acquire abdominal videos, facilitating the popularization of this technology. The optical flow vector of FM was coded in an HSL space, reducing the effect of ambient light and improving the quality of abdominal video images. We found the S signal was more accurate than the H signal in FM detection, which was possibly because the S signal was able to reflect the intensity of maternal abdominal movements. This capacity became greater, especially when FM was present, and so it has a better capacity to characterize the FM.

In this study, pregnant women were required to lie motionlessly during the abdominal video recording. However, maternal activity is inevitable during long-term monitoring. Therefore, many efforts have to focus on the automatic detection and removal of maternal interference in future studies. In addition to FM parameters, researchers could discriminate further between various FM activities such as kicking, stretching, and overall gross body movement using machine learning algorithms. Additionally, more volunteers in the second trimester, close to delivery, and with different outcomes will be recruited in future studies to obtain convincing results. The non-contact and contact method could be applied to pregnant women at the same time to compare their efficiency.

## 5. Conclusions

In this work, we first proposed a novel method for monitoring FM with abdominal videos recorded by a camera without touching the pregnant women and thus without inconvenience to them. The proposed method achieved a great performance, in which the FM parameters measured were in good agreement with the results manually labeled by the professionals. The changes in FM parameters with gestational week were consistent with pregnancy progress. The outline for the successful abdominal video-aided FM detection was presented, thereby paving the way for its application in a home-friendly environment in which the obtained FM parameters can be transmitted to clinicians via the Internet in the future. We conducted a feasibility study rather than an extensive clinical trial, and our efforts will be further validated in clinical practice.

## Figures and Tables

**Table 1:** FM parameters measured by different methods (median (25%, 75%)).

FM Parameters	Proposed Method		Manual Labeling
	H Signal	S Signal	
Number (times/h)	45.15 (23.08, 57.53)	62.51 (28.70, 69.10)	60.65 (28.18, 69.07)
Interval (s)	40.89 (37.75, 85.58)	50.55 (47.15, 99.33)	49.99 (48.92, 101.48)
Duration (s)	2.26 (1.89, 2.81)	1.62 (1.37, 1.83)	1.74 (1.49, 2.03)
Percentage (%)	3.19 (1.11, 4.17)	2.54 (1.04, 3.16)	2.78 (1.03, 3.26)

**Table 2:** Evaluation of the proposed method.

Proposed Method	TDR (%)	PPV (%)	SEN (%)	ACC (%)	F1_Score (%)
H signal	77.22	75.00	77.22	61.41	76.09
S signal	95.75	95.26	95.75	91.40	95.50

**Table 3:** Comparison results of FM recognition.

Research Team	Measurement	Algorithm	Gold Standard	Number of Subjects/ Recording GWs	TDR (%)	PPV (%)	SEN (%)	ACC (%)	F1_Score (%)
Proposed method	Camera	Optical flow	Manual labeling	5/28 to 36	96	95	96	91	96
Layeghy [34]	Accelerometry system	Time–frequency distribution and principal component analysis	Ultrasound and maternal perception	NA/NA	NA	95	92	92	93
Khlif [35]	Accelerometers for motion	Root-mean-square and time–frequency matched filters	Ultrasound	4/32, 32, 32, 35	80	77	NA	NA	NA
Liang [13]	Accelerometers	K-SVD dictionary learning and orthogonal matching pursuit algorithm	Maternal perception	4/NA	90	90	NA	NA	NA
Lai [37]	Acoustic sensors for vibration	Comb notch filtering and principal component analysis coordinate transform	Physician-identified	44/24 to 34	68	NA	NA	NA	NA
Rooijakkers [12]	Abdominal ECG recordings	Band-pass filtering and the R-peak detection algorithm	Ultrasound	20/22 to 40	NA	NA	64	68	NA
Schmidt [38]	Magneto-cardiographic	Moving correlation coefficient	Maternal perception	30/NA	NA	NA	81	NA	NA
Lu [39]	Fetal actography and tocography	Empirical mode decomposition, kohonen neural network and linear baseline estimation method	Physician-identified	52/NA	NA	71	82	NA	NA

NA: not available.

## Data Availability

The data are not publicly available due to privacy or ethical restrictions.
